# A Highly Pathogenic H5N1 Influenza A Virus Isolated from a Flamingo on the Caspian Sea Shore

**DOI:** 10.1128/MRA.00508-20

**Published:** 2020-09-24

**Authors:** Kobey Karamendin, Aidyn Kydyrmanov, Yermukhammet Kasymbekov, Klara Daulbayeva, Elizaveta Khan, Aigerim Seidalina, Marat Sayatov

**Affiliations:** a Scientific Production Center of Microbiology and Virology, Almaty, Kazakhstan; Queens College

## Abstract

In 2015, in the Kazakh part of the northern Caspian Sea region, during the monitoring of wild birds for avian influenza viruses, a highly pathogenic A/flamingo/Mangistau/6570/2015(H5N1) influenza virus was isolated from a dead flamingo. This study aimed to obtain the complete genome sequence of the isolate.

## ANNOUNCEMENT

Influenza A viruses are enveloped single-stranded RNA viruses, with a segmented negative-sense genome, that belong to the family *Orthomyxoviridae*, genus *Alphainfluenzavirus* (1). The highly pathogenic avian influenza (HPAI) virus of the H5N1 subtype continues to circulate in nature and is considered a severe epidemic threat.

**FIG 1:**
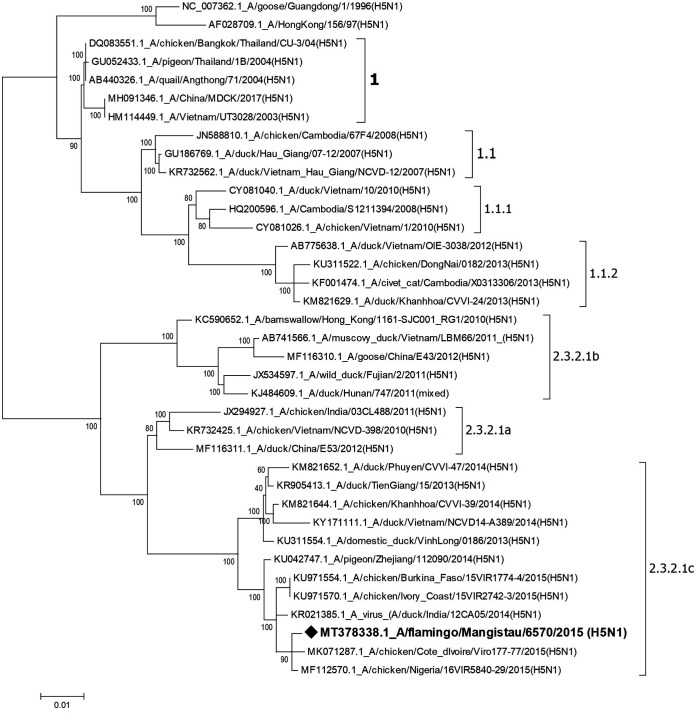
Phylogenetic tree of the HA gene of influenza A/H5N1 viruses of clade 2.3.2.1c and other viruses circulating around the globe.

The H5N1 influenza virus strain was isolated from a brain tissue specimen obtained from a dead flamingo at the Caspian Sea shore (44.47N, 51.17E). The sample was inoculated into 10-day-old embryonated chicken eggs and incubated at 37°C for 36 h. A hemagglutination assay with 0.75% chicken red blood cells was used to detect the virus in the allantoic fluid (2). Viral RNA was extracted from the allantoic fluid using the QIAamp viral RNA minikit (Qiagen, Germany) according to the manufacturer’s manual. Library preparation was conducted using the NEBNext Ultra RNA kit with the rRNA depletion kit (NEB, USA) according to the manufacturer’s protocol. Paired-end sequencing was performed on a MiSeq instrument using the reagent kit v3 (Illumina). Quality analysis of sequencing data was performed using FastQC (3). Default parameters were used for all software unless otherwise specified. In total, 2,168,460 raw sequencing reads with a mean length of 301 nucleotides and GC content of 55.4% were obtained. The sequence data obtained were trimmed at the 3′ and 5′ ends with an error probability limit of 0.05, assembled, and mapped with the Geneious installed mapper (medium sensitivity, four iterations) against influenza A/goose/Guangdong/1/1996(H5N1) reference sequences downloaded from GenBank (accession numbers AF144300 to AF144307) using Geneious Prime v2020.0.3 software (Biomatters, New Zealand) (4). The sequences obtained were aligned (Clustal W algorithm) and phylogenetic trees were constructed using the neighbor-joining method and the Tamura-Nei model (5) with MEGA 7.0 software (6), with 1,000 bootstrap replications to automatically assign confidence levels for the branches. The tree is drawn to scale, with branch lengths measured in the number of substitutions per site (Fig. 1).

The final obtained assembly contained 13,079 nucleotides, with a mean coverage of 3,383-fold. The deduced amino acid sequence of the hemagglutinin (HA) gene cleavage site revealed the presence of the PQRERRRKR*GLF motif that is typical of HPAI. BLASTn analyses showed significant genetic similarity of the flamingo isolate in all eight genes to the highly pathogenic H5N1 influenza viruses that were isolated from poultry in West Africa (Table 1) and caused outbreaks there (7). Phylogenetic analysis showed that the HA gene of the isolate was closely related to that of isolates from clade 2.3.2.1c, which has been identified since 2014 in Asia, the Middle East, Eastern Europe, and West Africa (Fig. 1). Supposedly, flamingos play some role in the intercontinental transfer of highly pathogenic variants of the avian influenza virus of the H5N1 subtype.

**TABLE 1:** Comparison of the nucleotide sequences of all genes of the influenza A/flamingo/Mangistau/6570/2015(H5N1) strain with the genetically closest strains in GenBank

Gene or segment	Size (nucleotides)	GC content (%)	Closest relative	Identity at nucleotide level (%)	GenBank accession no. for closest relative
PB2	2,280	45.2	A/chicken/Nigeria/16VIR5840-34/2015(H5N1)	99.61	MF112287
PB1	2,274	43.1	A/chicken/Nigeria/16VIR5840-25/2015(H5N1)	99.69	MF112377
PA	2,151	43.1	A/goose/Nigeria/16VIR5840-3/2015(H5N1)	99.67	MF112452
HA	1,704	40.8	A/chicken/Nigeria/16VIR5840-29/2015(H5N1)	99.65	MF112570
NP	1,497	48.0	A/chicken/Nigeria/16VIR5840-56/2015(H5N1)	99.73	MF112697
NA	1,350	43.9	A/chicken/Nigeria/16VIR5840-27/2015(H5N1)	99.48	MF112768
M	982	47.6	A/chicken/Nigeria/16VIR5840-68/2015(H5N1)	99.90	MF112909
NS	841	44.5	A/goose/Nigeria/16VIR5840-3/2015(H5N1)	99.76	MF112944

### Data availability.

The eight near-complete genome segments are available in GenBank under accession numbers MT378335 to MT378342. Raw sequence reads were deposited under BioProject accession number PRJNA628150.
